# The Association Between Pregnancy-Induced Hypertension and Neonatal Cerebral Metabolism, Hemodynamics, and Brain Injury as Determined by Physiological Imaging

**DOI:** 10.3389/fphys.2022.756386

**Published:** 2022-02-28

**Authors:** Ying Qi, Zixuan Lin, Hanzhang Lu, Pengfei Zhao, Yang Hou, Jian Mao

**Affiliations:** ^1^Department of Radiology, Shengjing Hospital of China Medical University, Shenyang, China; ^2^Department of Radiology, Johns Hopkins University School of Medicine, Baltimore, MD, United States; ^3^Department of Pharmacology, School of Pharmaceutical Sciences, China Medical University, Shenyang, China; ^4^Department of Pediatrics, Shengjing Hospital of China Medical University, Shenyang, China

**Keywords:** pregnancy-induced hypertension, PC MRI, TRUST MRI, CMRO_2_, CBF, OEF

## Abstract

Pregnancy-induced hypertension (PIH) is common and may affect maternal and children’s healthcare. However, the neurobiological status of neonates born from mothers with PIH has yet to be elucidated. The present study employed physiological imaging to investigate the association between maternal PIH and a number of neonatal health parameters, including cerebral metabolism, hemodynamics, and pathophysiological vulnerabilities. Following the acquisition of ethical approval, we recruited 16 neonates with maternal PIH and 22 normal neonates (non-PIH) as controls. All neonates underwent magnetic resonance imaging (MRI) of the brain. Phase-contrast (PC) MRI and T2-relaxation-under-spin-tagging (TRUST) MRI were performed to determine global cerebral blood flow, oxygen extraction fraction (OEF), and cerebral metabolic rate of oxygen (CMRO_2_). These physiological parameters were then compared between PIH neonates and controls. Linear regression analysis was performed to investigate the associations between maternal PIH and each of the physiological parameters. Receiver operating characteristic curves (ROCs) were used to determine whether maternal systolic blood pressure (SBP), diastolic blood pressure (DBP), and mean arterial pressure (MAP) which could facilitate the diagnosis of neonatal brain injuries. PIH neonates showed significantly lower OEF (25.5 ± 8.8% vs. 32.6 ± 7.3%, *P* = 0.01) and CMRO_2_ (29.7 ± 9.4 vs. 40.9 ± 15.0 μmol/100 g/min, *P* = 0.01) compared to the controls. Maternal blood pressure levels [PIH or non-PIH groups, each one standard deviation (SD) increase in SBP, DBP, and MAP, respectively] were negatively associated with OEF [regression coefficient (β) = −7.9, *P* = 0.007; β = −4.2, *P* = 0.004; β = −3.6, *P* = 0.02; β = −4.0, *P* = 0.008, respectively). Furthermore, each one SD increase in maternal DBP and MAP was negatively associated with CMRO_2_ (β = −4.7, *P* = 0.03; β = −4.4, *P* = 0.04, respectively). The areas under the curves (AUCs) with 95% confidence intervals (CIs) for maternal SBP, DBP, and MAP were 0.90 (0.80–0.97), 0.85 (0.73–0.97), and 0.89 (0.76–0.99), respectively. The AUC values for maternal SBP, DBP, and MAP indicated good diagnostic ability for identifying neonatal brain injuries. The present study demonstrated that maternal PIH may be associated with a lower oxygen extraction and lower cerebral metabolism in neonates.

## Introduction

Pregnancy-induced hypertension (PIH) is a substantial global public health issue for maternal and children’s healthcare (Visintin et al., 2010). PIH complicates approximately 6–10% of all pregnancies globally (Kintiraki et al., 2015). It is one of the trigger factors for maternal posterior reversible encephalopathy syndrome (PRES) and iatrogenic premature birth. PRES is a radio-clinical entity that is caused by an acute elevation of blood pressure above the upper limit of hemodynamic autoregulation (Ollivier et al., 2017). PRES is characterized by headache, alertness impairment, seizures, and visual disturbance, and can be accompanied by vasogenic edema in the white matter (Marrone et al., 2016). Iatrogenic premature birth, especially a birth at <34 weeks gestation, is known to have a higher risk of intrauterine growth restriction (IUGR) and lower birth weight (LBW) (Lu et al., 2018). Although most such premature newborns exhibit appropriate catch-up in terms of weight and height, they have an increased risk of brain damage (Martinussen et al., 2005), growth failure (Tchamo et al., 2016), motor difficulties, developmental coordination disorders or delays (Burns et al., 2009), vascular diseases in adulthood (Christian et al., 2013), and type 2 diabetes (Barker, 2003). Consequently, gaining a better understanding of the neurobiological status of neonates and its specific relationship with maternal blood pressure in pregnancy may provide important insights into neonatal pathophysiological vulnerabilities. Global cerebral blood flow (CBF), oxygen extraction fraction (OEF), and cerebral metabolic rate of oxygen (CMRO_2_) are recognized as important physiological indices. These variables are usually measured *via* computed tomography (CT) (Zebedin et al., 2013), positron emission tomography (PET) imaging (Luo et al., 2014), near-infrared spectroscopy (NIRS) (Kusaka et al., 2014; Goeral et al., 2017), or ^133^xenon clearance (Colditz et al., 1988). Because these procedures either involve radiation exposure, present difficulties when imaging deep brain tissues, or require exogenous tracers, their applications in neonatal brain imaging are limited. In addition, small brain volume, susceptibility to motion, and limited scan time cause more challenges in neonatal physiological imaging. Recent advances in magnetic resonance imaging (MRI) technologies have allowed CBF to be measured using phase-contrast (PC) MRI and cerebral oxygen extraction to be determined using T_2_-relaxation-under-spin-tagging (TRUST) MRI. CBF and OEF can be combined to allow for the calculation of global CMRO_2_ (Liu et al., 2014). Importantly, all of these physiological measurements can be accomplished in less than five minutes and are therefore suitable for the neonatal population (Qi et al., 2018; Shetty et al., 2019). Most neonatal brain injury research has focused on hypoxic ischemic encephalopathy (HIE) using NIRS (Wintermark et al., 2014) and on white matter lesions using PC MRI and TRUST MRI (Qi et al., 2018). Few neurobiological studies have been conducted on the effects of maternal PIH on cerebral oxygen metabolism and hemodynamics in neonates.

The present study used PC MRI and TRUST MRI to determine CBF, OEF, and CMRO_2_ in a group of neonates with maternal PIH and compared these parameters to a group of newborns without PIH. The study objective was to understand the association between maternal PIH and neonatal cerebral metabolism, hemodynamics, and brain injuries, thereby gaining insight into the pathophysiological processes involved.

## Materials and Methods

### Enrollment

The study was approved by the Shengjing Hospital’s Institutional Review Board (IRB) (No. 2016PS173J) and conducted in accordance with the Declaration of Helsinki (as revised in 2013). The IRB agreed to waive the requirement for written informed consent since the data were collected as part of a clinically indicated MRI. A total of 38 newborns were enrolled between April 2016 and March 2017. The participants were categorized into PIH and non-PIH groups. The inclusion criteria for the PIH group were as follows: (1) newborns with maternal PIH, (2) age of <10 days after birth, (3) stabilized vital signs and no requirement for ventilation, and (4) for better understanding the severity of PIH, mothers who had undergone a brain MR scan during the perinatal period. PIH included gestational hypertension, preeclampsia, or eclampsia. Gestational hypertension was defined as a systolic blood pressure (SBP) of >140 mmHg and/or diastolic pressure of >90 mmHg after week 20 of gestation. Preeclampsia was defined as hypertension with proteinuria. Eclampsia was defined as convulsions occurring in patients with preeclampsia (1). The inclusion criteria for the non-PIH group were as follows: (1) newborns without maternal PIH, (2) age of <10 days after birth, (3) stabilized vital signs and no requirement for ventilation, and (4) no abnormalities on MRI and laboratory results at birth. The exclusion criteria included congenital malformations, severe infection, or unusable MRI results. Two experienced neuroradiologists (YQ and QSW, with 10 years of experience) blinded to group data were responsible for scoring and diagnosing MRIs using an established method for preterm infants (Woodward et al., 2006). The scoring system for the composite white matter and gray matter scores consisted of white matter/gray matter signal abnormality, loss in the volume of periventricular white matter, the extent of any cystic abnormalities, ventricular dilatation, and thinning of the corpus callosum, each using a three-point scale (Supplementary Table 1).

### Magnetic Resonance Imaging Protocol

All MRI scans were acquired using a 3.0 Tesla MRI system that was equipped with a phased-array head coil (Intera Achieva; Philips Healthcare, Best, Netherlands). Neonates were sedated by a pediatrician *via* nasal administration of chloral hydrate (50 mg/kg). Axial T_1_-weighted spin-echo images/T_2_-weighted images were acquired with the following settings: repetition time (TR), 200/5000 ms; echo time (TE), 2.3/80 ms; section thickness, 5/5 mm; field of view (FOV), 180 mm × 150 mm × 90 mm; matrix size, 224 × 162/112 × 112; and scan time, 34.4/40.9 s.

### Determination of Cerebral Blood Flow

**FIGURE 1:**
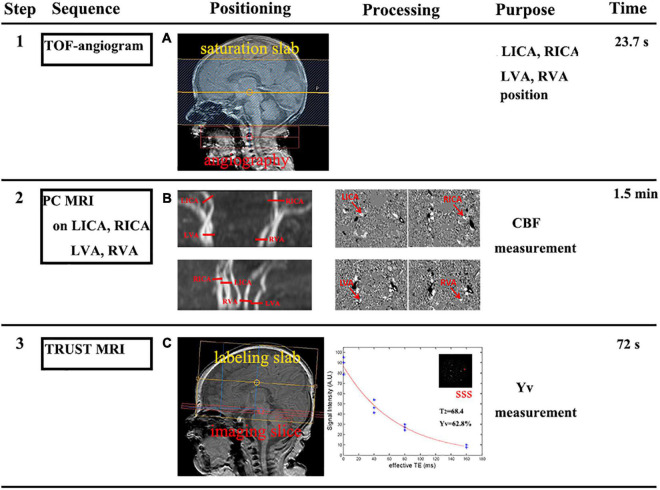
Framework showing how cerebral metabolic rate of oxygen (CMRO_2_) was measured (steps 1–3). **(A)** Step 1: a time-of-flight (TOF) MR angiogram (MRA) was performed to position the major feeding arteries of the brain, specifically the left and right internal carotid arteries (LICA and RICA) and the left and right vertebral arteries (LVA and RVA). **(B)** Step 2: slice positions of the PC MRI scans were overlayed on an angiogram image. Each red bar indicates the imaging slice of one PC MRI scan. Then, the manual delineation of the LICA, RICA, LVA, and RVA, was conducted to determine flux in these vessels. The sum of fluxes by brain weight yielded the cerebral blood flow (CBF). **(C)** Step 3: the imaging slice was positioned parallel to the intercommissural line with a 10 mm distance. TRUST MRI used a spin-labeling module to isolate pure venous blood signals in the superior sagittal sinus (SSS). Blood signals from the SSS were fitted to a monoexponential function of effective TE to yield blood T_2_; in turn, this can then be converted to Yv *via* a calibration plot.

First, a time-of-flight MR angiogram (MRA) was performed to allow visualization of the left and right internal carotid arteries and the left and right vertebral arteries. The imaging slab was positioned at the center of the epistropheus, with 60-mm saturation slabs placed above to suppress venous signals. The MRA parameters were as follows: TR, 20 ms; TE, 3.45 ms; flip angle, 18°; FOV, 90 mm × 90 mm × 20 mm; voxel size, 0.8 mm × 0.8 mm × 2 mm; and scan duration, 23.7 s. Second, PC MRI with bipolar gradients was used to encode the flow velocity of blood in the major feeding arteries, thereby providing a measurement of flow value. PC MRI was acquired with the following settings: single slice; voxel size, 0.5 mm × 0.5 mm × 3 mm; FOV, 180 mm × 180 mm × 3 mm; maximum velocity encoding, 20 cm/s (non-gated); averages, 2, total scan time, 1.5 min. Then, manual delineation of the four arteries was conducted on the magnitude image of each PC scan, and ROI masks were applied to the velocity maps to determine the flux in these vessels. The sum of fluxes in all vessels yielded blood flow to the whole brain (in mL/min). To convert this value to CBF, the brain volume was obtained using manual tracing of T_2_-weighted images while assuming a parenchymal density of 1.06 g/mL (Herscovitch et al., 1985). CBF was then calculated by dividing the total flux by brain weight (Figures 1A,B).

### Cerebral Metabolic Rate of Oxygen Calculation

Cerebral metabolic rate of oxygen was calculated based on Fick’s principle (Herscovitch et al., 1985; Liu et al., 2016); as shown in Equation 1. CMRO_2_ is expressed as μmol O_2_/100/min.


(1) $${\text{CMRO}}_{2}=\mathrm{CBF}\times \mathrm{OEF}\times 100\%\times \mathrm{Ya}\times \mathrm{Ca},$$

OEF could be determined by


(2) OEF=(Ya-Yv)/Ya×⁢100%,

where arterial oxygenation (Ya) and venous oxygenation (Yv) represented arterial and venous oxygenation, respectively. We used pulse oximeter to measure Ya on newborns according to previous literature (Liu et al., 2014). Pulse oximetry is designed to estimate peripheral oxygen saturation (SaO_2_). It gives a rough estimate of arterial blood oxygen content. The oximeter used in the Neonatal Oxygenation Prospective Meta-analysis trials had a reported accuracy of ±3% (1 SD) (Johnston et al., 2011; Cummings and Lakshminrusimha, 2017). Ya was measured on the neonatal right hand to gain a more accurate reflection of the SaO_2_ being delivered to the brain. Yv was measured using TRUST MRI according to previous literature (Lu and Ge, 2008; Liu et al., 2014). Lu and Ge (2008) reported that Yv measured by TRUST MRI could estimate venous oxygenation robustly with an estimation error of 1–2%. The parameters were as follows: labeling slab thickness, 80 mm; four effective TEs (eTE), 0, 40, 80, and 160 ms; TR, 3,000 ms; inversion time (TI), 1,022 ms; FOV, 160 mm × 160 mm × 5 mm; matrix size, 64 × 61; SENSE factor, 3; voxel size, 2.5 mm × 2.5 mm × 5 mm; τ_*CPMG*_, 10 ms; eTEs, three pairs; and scan time, 72 s. The imaging slice for TRUST MRI was based on the existing procedures (Qi et al., 2018). According to the principle that blood T_2_ has a calibration-able relationship with oxygenation level, TRUST MRI uses a spin-labeling module to isolate pure venous blood signals, employs a series of T_2_-preparation pulses to modulate the MRI signal, and utilizes the mono-exponential fitting of MRI signal to yield blood T_2_. Venous T_2_ was converted to Yv using a hematocrit (HCT)-specific T_2_-Yv calibration curve (Figure 1C; Liu et al., 2016). The PC and TRUST MRI data processing followed previously described procedures (Liu et al., 2014; Qi et al., 2018; Shetty et al., 2019). Ca is a parameter that can represent the blood’s oxygen-carrying capacity. Ca was based on each neonate’s individual HCT level and could be determined by


(3) $$\text{Ca}=\frac{\mathrm{HCT}\times 897}{44\%},$$

Ca is expressed as μmol O_2_/100 mL (Burgess et al., 2018).

### Statistical Analysis

Data normality was assessed using the Kolmogorov–Smirnov test, and the appropriate parametric or non-parametric test was applied. Continuous variables were represented as mean ± standard deviation (SD) or medians (interquartile range, total range). Categorical variables were described using frequencies (*n*, %). Student’s *t*-tests or Wilcoxon’s two-sample exact tests (for continuous variables), and Chi-squared tests (for categorical variables) were used to compare PIH (*n* = 16) and non-PIH (*n* = 22) neonates. The inter-rater variation for MRI scores determined by YQ and QSW was estimated using the intraclass correlation coefficient (ICC) and 95% confidence interval (CI). ICC agreement values were interpreted as slight (0.00–0.20), fair (0.21–0.40), moderate (0.41–0.60), substantial (0.61–0.80), and almost perfect (0.81–1.0) reliability. Multiple linear regressions were examined to predict the measured physiological parameters (OEF, CMRO_2_, CBF, and brain volume) that can be influenced by maternal PIH using three operating input parameters, including group (PIH or non-PIH), neonatal scan age, and birth weight. Similar analyses were used to examine the dependence of physiological parameters (OEF, CMRO_2_, CBF, and brain volume) on maternal blood pressure [SBP, diastolic blood pressure (DBP), and mean arterial pressure (MAP)]. The physiological parameters were assigned as the dependent variables, while scan age, birth weight, and maternal blood pressure were assigned as the independent variables. Considering that a one unit change in blood pressure had little effect on physiological and morphological parameters, multiple linear regression was performed, such that each SD increase in SBP, DBP, and MAP was considered as an independent variable. Receiver operating characteristic curves (ROCs) were used to determine whether maternal SBP, DBP, and MAP could facilitate the diagnosis of neonatal brain injuries (the range of MRI scores from 7 to 15). To identify the cut-off point for maternal blood pressure associated with neonatal brain injuries and the risk of neonatal pathophysiological vulnerabilities, the accuracy of a diagnostic test was classified using a traditional academic point system: excellent = 0.9–1.0, good = 0.8–0.9, fair = 0.7–0.8, poor = 0.6–0.7, and fail = 0.5–0.6. Linear regressions were examined to predict the measured physiological parameters (OEF, CMRO_2_, CBF, and brain volume) that can be influenced by MRI scores. All analyses were performed using SPSS software (version 21; IBM Corp., Armonk, NY, United States), where significance was set at *P* < 0.05.

## Results

### Basic Characteristics and Measurements of Physiological Parameters

**FIGURE 2:**
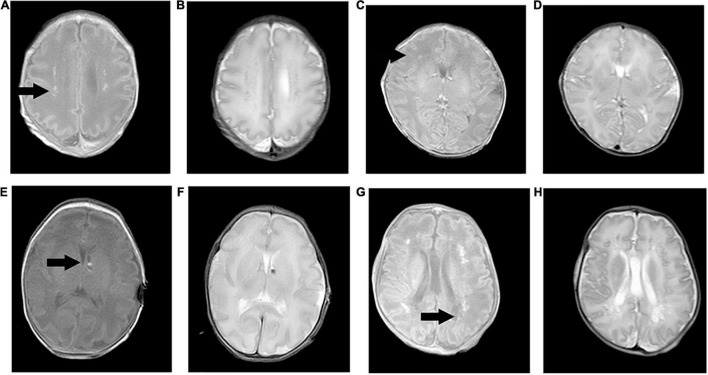
Pregnancy-induced hypertension cases with brain injuries on T_1_-weighted spin-echo images and T_2_-weighted images. **(A,B)** Case one (scan age, 34.1 weeks; birth weight, 2,240 g; male) showed bilateral and periventricular white matter damage with increased signal intensity on T_1_WI and decreased signal intensity on T_2_WI. **(C,D)** Case two (scan age, 36.6 weeks; birth weight, 2,010 g; female) had suffered a right frontal white matter injury with the same signal intensity. **(E,F)** Case three (scan age, 35.9 weeks; birth weight, 2,340 g; male) had clustered subependymal hemorrhages in the left interventricular foramen with slightly high signal intensity on T_1_WI and slightly low signal intensity on T_2_WI. **(G,H)** Case four (scan age, 37.9 weeks; birth weight, 1,560 g; male) had multiple white matter damage and *periventricular leukomalacia* in bilateral periventricular areas. Cystic lesions showed reduced signal intensity on T_1_WI, and increased signal intensity on T_2_WI.

**FIGURE 3:**
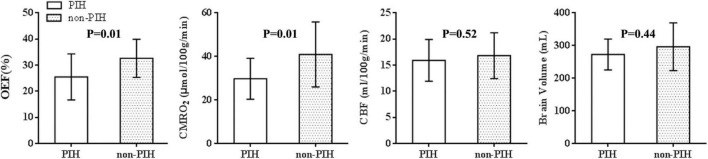
Oxygen extraction fraction-, CMRO_2_-, CBF-, and brain volume-data for 22 control and 16 maternal PIH infants. The mean, SD, minimum and maximum values for the OEF, CMRO_2_, CBF, and brain volume of the two groups were shown; the OEF and CMRO_2_, in PIH group differed significantly from the parameters in control group (*t* = –2.7, *P* = 0.01; *t* = –2.6, *P* = 0.01). There was no significant difference between the two groups with regards to CBF (*t* = –0.7, *P* = 0.52) or brain volume (*t* = –1.3, *P* = 0.22).

**FIGURE 4:**
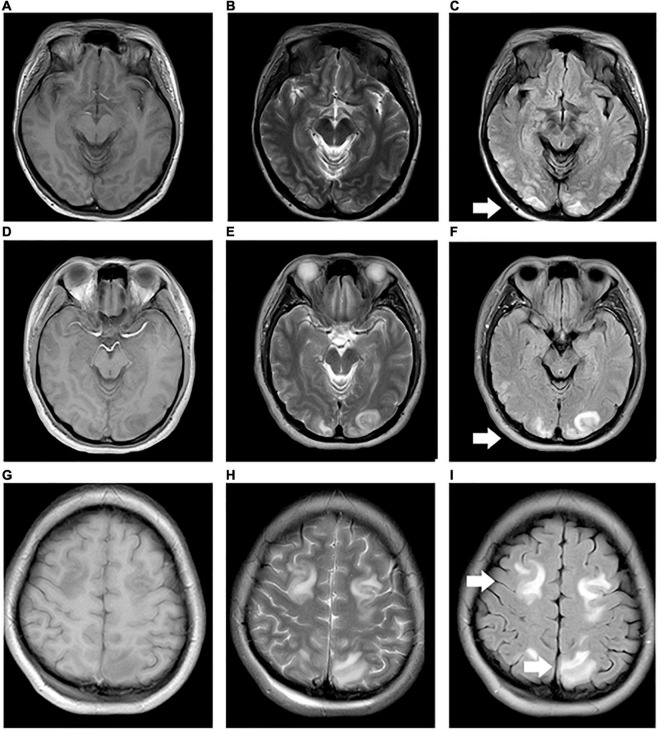
Maternal preversible encephalopathy syndrome. **(A–I)** Maps showing 33, 28, and 24 year-old pregnant women (BH: 200/130, 160/110, and 180/110 mmHg) with decreased signal intensity on T_1_WI, and increased signal intensity on T_2_WI and FLAIR in bilateral occipital regions, bilateral occipital and right temporal regions, and bilateral parietal and frontal regions, respectively.

Table 1 shows the demographic data, neonatal clinical characteristics, physiological parameters, and maternal characteristics. The prenatal characteristics of the PIH group featured six (37.5%) cases with antepartum hemorrhage, four (25%) cases with premature membrane rupture, four (25%) cases with an umbilical cord around the neck or disappearance of the umbilical blood flow, and two (12.5%) cases with oligohydramnios. Brain MRI results revealed four (25%) newborns with white matter damage (WMD), two (12.5%) newborns with subependymal hemorrhage, and two (12.5%) newborns with WMD and *periventricular leukomalacia* (PVL) (Figure 2). The ICC for the MRI scores was 0.98 (95% CI: 0.97–0.99). The reliability was considered to be almost perfect. In comparison with the non-PIH neonates, the PIH neonates showed significantly lower OEF (25.5 ± 8.8 vs. 32.6 ± 7.3%, *P* = 0.01) and CMRO_2_ (29.7 ± 9.4 vs. 40.9 ± 15.0 μmol/100 g/min, *P* = 0.01). There was no significant difference between the two groups with regards to CBF (15.9 ± 4.0 vs. 16.8 ± 4.4 mL/100 g/min, *P* = 0.52) or brain volume (289.0 ± 44.2 vs. 314.1 ± 77.6 mL, *P* = 0.44). The mean and SD for OEF, CMRO_2_, CBF, and brain volume from both groups were shown by means of boxplots in Figure 3. Maternal SBP (*P* < 0.001), DBP (*P* < 0.001), and MAP (*P* < 0.001) were significantly higher in the PIH than in the non-PIH group. There were three (18.8%) neonates in the PIH group with maternal PRES who exhibited reduced signal intensity on T_1_-weighted spin-echo images, increased signal intensity on T_2_-weighted images, and fluid-attenuated inversion recovery (FLAIR) images in bilaterally occipital regions and/or parietal, temporal, or frontal regions (Figure 4).

**TABLE 1:** Baseline characteristics of neonates with maternal PIH and controls.

Variables	PIH (*n* = 16)	Non-PIH (*n* = 22)	*P*
**Neonatal characteristics**			
Males, *n* (%)	10 (62.5)	18 (81.8)	0.41
Birth weight, g	1863.2 ± 312.3	2334.4 ± 1090.3	0.07
Birth age, weeks	34.5 ± 1.3	34.2 ± 4.5	0.75
Scan age, weeks	34.9 ± 2.1	36.6 ± 3.5	0.07
One-minute Apgar scores	8.3 ± 1.6	8.2 ± 2.1	0.88
Five-minute Apgar scores	8.8 ± 1.6	9.6 ± 0.7	0.08
**Neonatal blood gas analysis**			
Total tested, *n* (%)	14 (87.5)	20 (90.9)	
pH	7.4 ± 0.05	7.4 ± 0.04	0.91
PCO_2_, mmHg	40.3 ± 5.7	43.4 ± 7.0	0.15
PaO_2_, mmHg	84.9 ± 10.0	88.9 ± 7.5	0.17
SaO_2_, %	97.8 ± 1.9	97.0 ± 2.3	0.27
Glucose, mmol/L	4.6 ± 0.5	4.9 ± 0.6	0.07
**Physiological parameters**			
OEF, %	25.5 ± 8.8	32.6 ± 7.3	0.01*
CMRO_2,_ μmol/100 g/min	29.7 ± 9.4	40.9 ± 15.0	0.01*
CBF, mL/100 g/min	15.9 ± 4.0	16.8 ± 4.4	0.52
Brain volume, mL	289.0 ± 44.2	314.1 ± 77.6	0.44
**Maternal characteristics**			
Primiparity, *n* (%)	8 (50.0)	15 (68.2)	0.14
Maternal age, year	27.8 ± 4.2	29.3 ± 3.5	0.21
SBP, mmHg	173.7 ± 13.1	116.6 ± 7.2	<0.001*
DBP, mmHg	112.3 ± 7.8	80.0 ± 4.8	<0.001*
MAP, mmHg	132.8 ± 9.2	91.8 ± 3.4	<0.001*

Data expressed as n (% total) or mean ± SD. P-values were based on Chi-squared tests and Student’s t-tests. *P < 0.05.

*PIH, pregnancy-induced hypertension; PCO_2_, partial pressure of carbon dioxide; PaO_2_, partial pressure of oxygen; SaO_2_, oxygen saturation; OEF, oxygen extraction fraction; CMRO_2_, cerebral metabolic rate of oxygen; CBF, cerebral blood flow; SBP, systolic blood pressure; DBP, diastolic blood pressure; MAP, mean arterial pressure.*

### Association Between Maternal Pregnancy-Induced Hypertension (Group, Systolic Blood Pressure, Diastolic Blood Pressure, or Mean Arterial Pressure per SD) and Measured Physiological Parameters

As shown in Tables 2–5, maternal blood pressure parameters (group, SBP, DBP, and MAP per SD) were all negatively associated with OEF (β = −7.9, *P* = 0.007; β = −4.2, *P* = 0.004; β = −3.6, *P* = 0.02; β = −4.0, *P* = 0.008, respectively), although SBP was not associated with CMRO_2_ (β = −3.8, *P* = 0.07). Maternal blood pressure parameters (group, SBP, DBP, and MAP per SD) were not associated with CBF or brain volume (*P* > 0.05). Furthermore, scan age was positively associated with CMRO_2_ (regression coefficient (β) = 1.8, *P* = 0.03; β = 1.7, *P* = 0.04; β = 1.6, *P* = 0.04; β = 1.7, *P* = 0.04), CBF (β = 0.9, *P* = 0.001; β = 0.9, *P* = 0.001; β = 0.8, *P* = 0.001; β = 0.9, *P* = 0.001) and brain volume (β = 15.7, *P* < 0.001; β = 15.5, *P* < 0.001; β = 15.5, *P* < 0.001; β = 15.6, *P* < 0.001). Birth weight showed no dependence on OEF, CMRO_2_, CBF, or brain volume (*P* > 0.05).

**TABLE 2:** Association between maternal PIH (PIH and non-PIH group) and measured physiological parameters (*N* = 38).

Factor	β (95% CI)			*P*-value		
		
	Scan age	Birth weight	Group	Scan age	Birth weight	Group
OEF, %	0.2 (−0.9, 1.3)	−0.002 (−0.006, 0.001)	−7.9 (−13.5, −2.3)	0.67	0.20	0.007*
CMRO_2_, μmol/100 g/min	1.8 (0.2, 3.4)	0.001 (−0.005, 0.007)	−7.6 (−15.9, 0.7)	0.03*	0.73	0.07
CBF, mL/100 g/min	0.9 (0.4, 1.4)	−0.001 (−0.003, 0.001)	0.2 (−2.4, 2.7)	0.001*	0.31	0.89
Brain volume, mL	15.7 (11.1, 20.4)	0.02 (0, 0.03)	9.4 (−14.8, 33.5)	<0.001*	0.05	0.44

*β, regression coefficient; CI, confidence interval; OEF, oxygen extraction fraction; CMRO2, cerebral metabolic rate of oxygen; CBF, cerebral blood flow.*

*P < 0.05.

**TABLE 3:** Association between maternal SBP (mmHg) and measured physiological parameters (*N* = 38).

Factor	β (95% CI)			*P*-value		
		
	Scan age	Birth weight	SBP/SD	Scan age	Birth weight	SBP/SD
OEF, %	0.2 (−0.9, 1.2)	−0.002 (−0.006, 0.001)	−4.2 (−7.0, −1.4)	0.76	0.21	0.004*
CMRO_2_, μmol/100 g/min	1.7 (0.1, 3.3)	0.001 (−0.005, 0.007)	−3.8 (−8.0, 0.4)	0.04*	0.70	0.07
CBF, mL/100 g/min	0.9 (0.4, 1.4)	−0.001 (−0.003, 0.001)	−0.1 (−1.4, 1.2)	0.001*	0.29	0.88
Brain volume, mL	15.5 (10.7, 20.2)	0.02 (−0.001, 0.03)	−0.2 (−12.5, 12.1)	<0.001*	0.07	0.98

*β, regression coefficient; SBP, systolic blood pressure; SBP/SD, per one SD increase of SBP; CI, confidence interval; OEF, oxygen extraction fraction; CMRO_2_, cerebral metabolic rate of oxygen; CBF, cerebral blood flow.*

*P < 0.05.

**TABLE 4:** Association between maternal DBP (mmHg) and measured physiological parameters (*N* = 38).

Factor	β (95% CI)			*P*-value		
		
	Scan age	Birth weight	DBP/SD	Scan age	Birth weight	DBP/SD
OEF, %	0.1 (−1.0, 1.3)	−0.002 (−0.006, 0.002)	−3.6 (−6.6, −0.7)	0.80	0.24	0.02*
CMRO_2_, μmol/100 g/min	1.6 (0.1, 3.2)	0.001 (−0.005, 0.006)	−4.7 (−8.9, −0.5)	0.04*	0.73	0.03*
CBF, mL/100 g/min	0.8 (0.4, 1.3)	−0.001 (−0.003, 0.001)	−0.3 (−1.6, 1.0)	0.001*	0.27	0.65
Brain volume, mL	15.5 (10.9, 20.5)	0.02 (−0.001, 0.03)	2.7 (−9.9, 15.2)	<0.001*	0.06	0.67

*DBP, diastolic blood pressure; SD, standard deviation; DBP/SD, per one SD increase of DBP; CI, confidence interval; OEF, oxygen extraction fraction; CMRO_2_, cerebral metabolic rate of oxygen; CBF, cerebral blood flow.*

*P < 0.05.

**TABLE 5:** Association between maternal MAP (mmHg) and measured physiological parameters (*N* = 38).

Factor	β (95% CI)			*P*-value		
		
	Scan age	Birth weight	MAP/SD	Scan age	Birth weight	MAP/SD
OEF, %	0.1 (−1.0, 1.2)	−0.002 (−0.006, 0.001)	−4.0 (−6.8, −1.1)	0.80	0.22	0.008*
CMRO_2_, μmol/100 g/min	1.7 (0.06, 3.3)	0.001 (−0.005, 0.007)	−4.4 (−8.5, −0.2)	0.04*	0.72	0.04*
CBF, mL/100 g/min	0.9 (0.4, 1.4)	−0.001 (−0.003, 0.001)	−0.2 (−1.5, 1.1)	0.001*	0.28	0.75
Brain volume, mL	15.6 (10.8, 20.3)	0.02 (−0.001, 0.03)	1.3 (−11.1, 13.8)	<0.001*	0.06	0.83

*MAP, mean arterial pressure; SD, standard deviation; MAP/SD, per one SD increase of MAP; CI, confidence interval; OEF, oxygen extraction fraction; CMRO_2_, cerebral metabolic rate of oxygen; CBF, cerebral blood flow.*

*P < 0.05.

### Receiver Operating Characteristic Curves for the Diagnosis of Neonatal Brain Injuries

**FIGURE 5:**
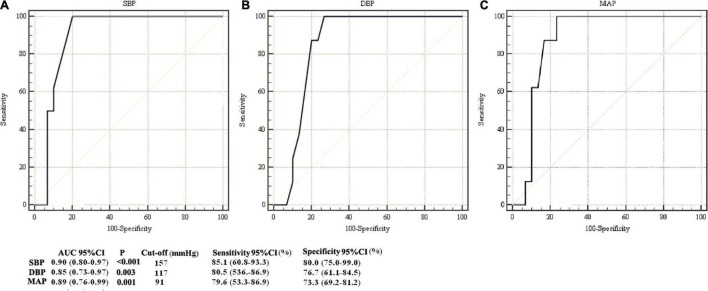
Comparison of ROC curves in identifying newborns with abnormal structural MRI. ROCs for analysis of the association between maternal systolic blood pressure (SBP) and neonatal brain injury on structural MRI **(A)**, between maternal diastolic blood pressure (DBP) and neonatal brain injury on structural MRI **(B)**, and between maternal mean arterial pressure (MAP) and neonatal brain injury on structural MRI **(C)**. The areas under curves (AUCs) for SBP, DBP and MAP were 0.90 [confidence interval (CI): 0.80–0.97], 0.85 (0.73–0.97), and 0.89 (0.76–0.99), respectively.

Receiver operating characteristic curves were used to evaluate and analyze the association between maternal blood pressure parameters (SBP, DBP, and MAP) and neonatal brain injuries (Figure 5). Areas under the curves (AUCs) with 95% CIs for maternal SBP, DBP, and MAP were 0.90 (0.80–0.97), 0.85 (0.73–0.97), and 0.89 (0.76–0.99), respectively. The AUC values for maternal SBP, DBP, and MAP indicated good diagnostic utility for identifying neonatal brain injuries. The cut-off values for SBP, DBP, and MAP were 157, 117, and 91 mmHg, respectively. Sensitivities with 95% CIs were 85.1% (60.8–93.3%), 80.5% (53.6–86.9%), and 79.6% (53.3–86.9%), respectively. Specificities with 95% CIs were 80.0% (75.0–99.0%), 76.7% (61.1–84.5%), and 73.3% (69.2–81.2%), respectively.

### Association Between Magnetic Resonance Imaging Scores and Measured Physiological Parameters

Magnetic resonance imaging scores were all negatively associated with OEF (β_*YQ*_ = −1.5, *P* < 0.001; β_*QSW*_ = −1.5, *P* < 0.001, respectively) and CMRO_2_ (β_*YQ*_ = −1.4, *P* = 0.02; β_*QSW*_ = −1.5, *P* = 0.01, respectively), although MRI scores were not associated with CBF (β_*YQ*_ = −0.009, *P* = 0.96; β_*QSW*_ = −0.04, *P* = 0.83, respectively) or brain volume (β_*YQ*_ = 1.5, *P* = 0.62; β_*QSW*_ = 1.7, *P* = 0.56, respectively).

## Discussion

The principal finding of the present study was that neonates with maternal PIH generally had lower metabolism and oxygen extraction (i.e., higher Yv). Maternal blood pressure was found to be a significant feature associated with neonatal neurodevelopmental dysplasia.

### Effect of Maternal Pregnancy-Induced Hypertension on Neonates

Throughout the course of the pregnancy, fetuses gradually mature and their cerebral vascular diameters increase. The CBF and CMRO_2_ also increase accordingly. In the pathological state of PIH, the maternal systemic arteriole spasm leads to the placental microvascular spasm. This results in the abnormal placentation, which is characterized by a shallow placental invasion of the decidua and abnormal uterine spiral artery remodeling (James et al., 2012). The abnormal placentation compromises the uterine blood flow at the expense of the growing placenta and fetus. At this moment, the cerebral tissue undergoes a compensatory period, while the fetal cerebral vessels can still experience autoregulation. As the cerebral perfusion pressure decreases, the cerebral vessels contract and the blood flow is redistributed to maintain cerebral perfusion. As expected, the PIH group showed reduced CMRO_2_ and OEF. There was no significant difference between the two groups with regards to CBF (15.9 ± 4.0 vs. 16.8 ± 4.4 mL/100 g/min, *P* = 0.52). Although there are no abnormal changes in the histo-morphology of the neonatal brain with maternal PIH, continuously abnormal hemodynamics might lead to chronic hypoxia or neurodevelopmental delay. Half of the newborns (8/16, 50%) in the PIH group showed a normal structural MRI result. However, the normal morphological status observed in the brain MRIs of the PIH group did not equate to normal physiology in terms of cerebral metabolism and hemodynamics. Chronic ischemia and hypoxia might increase the risks of adverse pregnancy outcomes, including fetal IUGR, preterm birth, iatrogenic premature delivery, and LBW (Odell et al., 2006; Burton et al., 2009; Misra et al., 2009; Kintiraki et al., 2015; Lu et al., 2018). In addition, PIH neonates in the present study were all preterm and with LBW (16/16, 100%). Hypotension with low blood flow and ischemia-reperfusion is known to contribute to the pathogenesis of brain injury (Rhee et al., 2018). In a previous study, Laptook et al. (2005) have proposed that premature status and LBW should be considered as risk factors for neurodevelopmental disorders, regardless of whether they were accompanied by normal head ultrasound or MRI results during the neonatal period. Furthermore, when the blood pressure of pregnant women increases further, their umbilical vascular permeability also increases. This results in particular aggravation of fetal ischemia and hypoxia, leading to hypercapnia and the accumulation of lactic acid. Since cerebral capillary vessel endothelial cells have three to five times as many mitochondria as other vascular endothelial cells in the body, the stimulation of hypoxia and acidic metabolites can easily lead to cerebral capillary damage, autonomic regulation dysfunction, passive vasodilatation, and increase in perfusion (Odell et al., 2006). When entering into the decompensation, impaired cerebral vascular autoregulation cannot maintain the fetal cerebral perfusion, showing lower blood supply and oxygen metabolism. The aggravation of hypoxia and dramatic fluctuation of blood pressure can cause the cerebral vessels to rupture easily, thus leading to morphological damage. Furthermore, the preterm neonates exhibit fragile periventricular vascular overgrowth in the germinal matrix and underdeveloped vascular networks. This might increase the risk of hemorrhages, leukoencephalopathy, and PVL (Rhee et al., 2018). We speculated that maternal PIH might play a role in the pathogenic process of relevant neonatal morbidities. This explained why maternal blood pressure was associated with neonatal neurodevelopmental dysplasia. Reviewing our PIH group, half of the newborns (8/16, 50%) showed a normal structural MRI result. The lack of decompensated cases of the PIH group might be the reason why OEF and CMRO_2_ were affected while CBF was not affected by maternal PIH. These regrets and omissions should be the direction for future research.

### Effect of Pregnancy-Induced Hypertension on Pregnant Women

Pregnancy-induced hypertension also has adverse effects on pregnancy outcomes. It was found that the PIH mothers all had abnormal manifestations, which is common and typical in PIH (Marrone et al., 2016). The present PIH group included three infants (3/16, 18.8%) with maternal PRES. We propose that the involved pathophysiological mechanism is related to cerebral autoregulation. When blood pressure exceeds the cerebrovascular autoregulatory limits, it might lead to unrestrained vasodilation followed by endothelial damage, disruption of the blood–brain barrier, and vasogenic edema (Laptook et al., 2005; Marrone et al., 2016). PRES was often located in the occipital regions on the MRI. This can be explained by the special anatomical-physiological differences between the anterior and posterior circulation. The anterior circulation involves the internal carotid artery, while the posterior circulation involves the vertebral basilar artery. With a higher density of autonomic innervation, the anterior circulation can better control the cerebral autoregulation. The posterior circulation is more vulnerable to abrupt blood pressure variations and blood-brain barrier disruption. Later in life, mothers with PIH (especially preeclampsia) have a two- to fourfold higher risk of stroke (Bushnell and Chireau, 2011; Marrone et al., 2016; Cunningham and LaMarca, 2018). Future research is needed to investigate the effects of gestational hypertension on brain injuries in pregnant women, the underlying pathophysiology of blood-brain barrier permeability, the loss of cerebral vascular autoregulation, and cerebral hemodynamic changes.

Cerebral blood flow, OEF, and CMRO_2_ can be measured using PC and TRUST MRI and have previously been reported in healthy and HIE patients (Liu et al., 2014; Shetty et al., 2019). The physiological parameters measured by Liu et al. (2014) in newborns at term with normal structural MRI results (CMRO_2_: 38.3 ± 17.7 μmol/100 g/min, OEF: 33.3 ± 2.7%) were generally in agreement with the physiological parameters measured in the non-PIH group (CMRO_2_: 40.9 ± 15.0 μmol/100 g/min, OEF: 32.6 ± 7.3%). These results demonstrated the accuracy and reliability of PC and TRUST MRI in the assessment of neonatal cerebral hemodynamics and metabolism.

Cerebral metabolic rate of oxygen and CBF, as well as morphological indices of brain volume, all increased with scan age. These positive correlations indicated that with growth and development, the oxygen metabolism and blood supply in the brain increase to meet the escalating energy demand. These results were consistent with previous studies in that CMRO_2_ and CBF were indirect parameters associated with brain development (Liu et al., 2014; Qi et al., 2018). The association between MRI scores and physiological parameters (OEF and CMRO_2_) suggested that physiological imaging might be helpful to quantitatively reflect brain maturity and injury severity.

Few studies have investigated the relationship between maternal PIH and oxygen delivery, metabolism, or brain injuries in the offspring. Unlike previous literature that reported that maternal MAP showed a linear positive relationship with their own brain oxygenation (Sekhon et al., 2019), or that neonatal MAP waves were negatively correlated with changes in NIRS-measured cerebral blood volume (when autoregulation was intact) (Rhee et al., 2018), the present study outcomes showed that maternal PIH was associated with neonatal brain injuries. Each one SD increase in maternal blood pressure was negatively associated with OEF and negatively associated with neonatal CMRO_2_. This suggested that increased maternal blood pressure might be associated with neurodevelopmental delay in neonates. The reduced brain oxygen intake in offspring led to a reduction in brain oxygen metabolism. This might be related to the autoregulation of premature cerebral hemodynamics. CBF regulation in neonatal brain is distinct from that of the fully matured brain. Infants born prematurely have an anatomically incomplete and underdeveloped cerebral vasculature and cannot fully auto-regulate. This has implications for many preterm infants during the third trimester (Rhee et al., 2016). Investigating the effects of maternal PIH on neonatal cerebral metabolism, hemodynamics, and brain injury might aid clinicians in neurophysiological profiling, timely diagnosis, treatment, and providing a better prognosis of at-risk brain damage in newborns with maternal PIH.

### Limitations

There were only 38 cases in the two groups due to the need for brain MR scans for both neonates and their mothers, which was a limitation in the present study. In future research, the scope of the study should be broadened to include follow-up of neonates with maternal PIH and their mothers, clarifying the impact of increased blood pressure on the offspring’s neurocognitive deficits. Another limitation was a birth age of less than 10 days. The observation time was too short. In the future, we will collect and group offspring by age, and increase the cognitive examination, language, social-emotional development, and behavior evaluation for more rigorously and scientifically study. Subject recruitment should also be increased to further stratify the study population by blood pressure and offspring’s sex. A multicenter approach should also be fostered in order to ascertain neurocognitive thresholds for the increase in MAP and decrease in CMRO_2_. Large sample data should be collected which may have more clinical value in quantifying brain maturity and injury severity. Compared with the accurate arterial and venous markers oxygenation, there were errors with Ya and Yv measured by pulse oximeter and TRUST MRI. This mis-match might affect our results. However, the comparison between the PIH group and the non-PIH group was still valid since these two groups were evaluated under the same measurement condition. Near-infrared spectroscopy (NIRS) is used as a measure of cerebral tissue oxygen saturation. In future work, we will try to use NIRS, in conjunction with pulse oximetry, to get a more accurate reflection of the SaO_2_ being delivered to the brain.

## Conclusion

The results of the present study demonstrated that maternal PIH may be associated with lower cerebral metabolism and oxygen consumption in neonates. Maternal blood pressure is a significant feature that was also associated with neonatal neurodevelopmental dysplasia.

## Data Availability Statement

The raw data supporting the conclusions of this article will be made available by the authors, without undue reservation.

## Ethics Statement

The studies involving human participants were reviewed and approved by the Shengjing Hospital’s Institutional Review Board (IRB) (No. 2016PS173J). Written informed consent from the participants’ legal guardian/next of kin was not required to participate in this study in accordance with the national legislation and the institutional requirements. Written informed consent was obtained from the individual(s), and minor(s)’ legal guardian/next of kin, for the publication of any potentially identifiable images or data included in this article.

## Author Contributions

YQ conceived the idea, designed the study, and drafted and edited the manuscript. ZL provided post-processing assistance. HL provided the pulse sequence and participated in the interpretation of the data. PZ contributed to analysis of the data. JM participated in the enrollment of patients and controls. YH provided administrative support. All authors have read and approved the final manuscript for publication.

## Conflict of Interest

The authors declare that the research was conducted in the absence of any commercial or financial relationships that could be construed as a potential conflict of interest.

## Publisher’s Note

All claims expressed in this article are solely those of the authors and do not necessarily represent those of their affiliated organizations, or those of the publisher, the editors and the reviewers. Any product that may be evaluated in this article, or claim that may be made by its manufacturer, is not guaranteed or endorsed by the publisher.
