# Supplementary material for: The Association Between Pregnancy-Induced Hypertension and Neonatal Cerebral Metabolism, Hemodynamics, and Brain Injury as Determined by Physiological Imaging
Source: Front Physiol. 2022 Feb 28;13:756386. doi: 10.3389/fphys.2022.756386 (PMC8918661; doi:10.3389/fphys.2022.756386)
Supplement: Supplementary file 1 [file Table_1.docx]

**Supplementary TABLE 1. Scoring white and gray matter abnormalities on MRI in neonates**

| **Grade** | **White matter and gray matter scores** | | | | | **Total scores** |
| --- | --- | --- | --- | --- | --- | --- |
|  | Signal abnormality in per hemisphere | Loss in periventricular white matter | Extent of cysts | Ventricular dilatation | Corpus callosum |  |
| 1 | normal signal | normal volume associated with small ventricles | no cyst | no ventricular dilatation | normal |  |
| 2 | focal high signal (≤2 regions ) | mild reduction with increased ventricular size | ≤2 mm single focal cyst | moderate enlargement | focal thinning |  |
| 3 | multiple high signal (> 2 regions) | marked reduction with marked increase in the size of the ventricle and/or extra-axial space | multiple cysts or a single larger (>2mm) | global enlargement including significant enlargement of all horns | global thinning |  |
| Total scores | Categorized as:  a) no abnormality - Total score 5 to 6  b) mild abnormality – Total score 7 to 9  c) moderate abnormality – Total score 10 to 12  d) severe abnormality – Total score 13 to 15 | | | | |  |
